# Supplementary material for: Learning curve and surgical outcome of robotic assisted colorectal surgery with ERAS program
Source: Sci Rep. 2022 Nov 29;12:20566. doi: 10.1038/s41598-022-24665-w (PMC9709162; doi:10.1038/s41598-022-24665-w)
Supplement: Supplementary file 2 — Supplementary Information 2. [file 41598_2022_24665_MOESM2_ESM.zip › RAL Raw data-Ñ[▒K/073/OP N_ñw╝╨░O▒Kñσ.pdf]

W52 / W52

臺中榮民總醫院
手術室護理記錄

[redacted]

基本資料

- 室別： 開刀房-17室，手術科別： CRS，手術類別： 常規，日期： 2021/04/09
- 進入時間： 1920 ； 麻醉開始時間： 1950 ； Time Out時間： 2146 ； 劃刀時間： 2147 ； 手術結束時間： 0115 送出時間： 0145 ；
- 麻醉方式： GE
  診斷：Malignant tumor of A-colon
  手術名稱：Robot Assisted Colectomy(R)
  手術局部用藥：nil
  術前預防性抗生素：
  - 抗生素名稱一： Veterin 1GM IVD由麻醉護理師執行
  - 時間： 2040
  - 上次簽章: 賴芸
  ，追加預防性抗生素：
  - 抗生素名稱一： Veterin 1GM IVD由麻醉護理師執行
  - 時間： 0040
  - 上次簽章: 張玉梅
- 手術醫師： 蔣鋒帆，林陳彥，江世偉
- 麻醉醫師： VS 劉恆豪
-
  - 刷手護士A ： 蘇敬涵，時間： 1920，刷手護士B ： 陳妍希，時間： 1920，接替刷手一： 彭芳渝/張玉梅，時間： 2000，接替刷手二： 邱欐媃，時間： 2300
  - 流動護士A ： 賴芸，時間： 1920，接替流動一： 張玉梅，時間： 2200，接替流動二： 胡穎汝，時間： 0030

皮膚狀況

A-V Shunt： 無
術前： 完整
記錄者： 上次簽章: 賴芸
術後： 完整
記錄者： 胡穎汝

手術準備

手術臥式 單一手術臥式

-
  - 手術臥式： 仰臥
  - 使用附件： 溫毯 38 ℃，頭圈
  - 皮膚準備： 10%Aq Betadine，Hibiscrub
  - 電燒： 使用電燒: 電極片位置： 左大腿，記錄者： 上次簽章: 賴芸

止血帶T： 無

植入物

- 植入物名稱/號碼/數量/部位：Covidien GIA 80-3.8*2/Colon

，記錄者： 上次簽章: 張玉梅

標本送檢

- 細菌培養 無
- 細胞學 無
- 冰凍切片 無
- 病理標本
  組織名稱一：Ileocolic LN
  ，
  組織名稱二：A-Colon

- 截肢 無

標本交付 標本： 1 袋 ， 標本： 0 盒 ， 標本： 1 瓶 ， 細胞學： 0 管 ， Culture Tube： 0 管 ， 被交付醫師： CR 林陳彥 ，

- 記錄者： 胡穎汝
- 時間： 0145

傷口術後敷料

傷口敷料： 傷口敷料： 無

- 請於圖上標示部位：

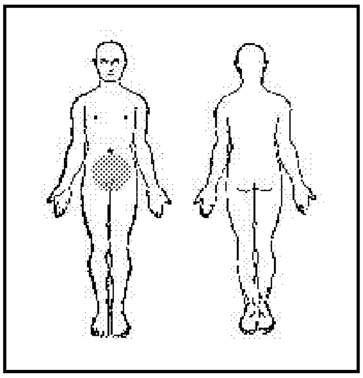

- 傷口敷料： Menpore

導管

導管	天數	導管資訊	插入日期	插入時間	更換日期	拔除日期	拔除時間	拔除原因	預計拔除/更換	預計拔除/更換日期
Foley(1)	2	號碼:16 Fr.; 部位:膀胱;	2021/04/09	2000						
Epidural PCA(1)	2	部位:脊髓;	2021/04/09	2000						
Double Lumen CVC(1)	2	號碼:8 fr; 深度:15cm; 部位:jugular (右); 象限:RU;	2021/04/09	2030						
A-line (1)	2	號碼:20 G; 部位:LT HAND;	2021/04/09	2000						

用物計數

- 手術中出血量： minimal c.c.
- 手術中尿量： 700 c.c.
-
  - 其它： 無

用物計數： 多切口同時縫合

- 1.器械： 正確 ，2.縫針： 正確 ，3.刀片： 正確 ，4.紗布： 正確 ，5.腹部墊： 未使用 ，6.紗球： 未使用 ，7.PEANUTS ： 未使用 ，8.紗條： 未使用 ，9.絲帶： 未使用 ，10.針頭： 未使用 ，11.Vessel loop： 未使用 ，12.Boots： 未使用 ，13.Jelco： 未使用 ，14.棉條： 未使用 ，
  - 第一次：
    - 刷手護士 ： 邱權婇/胡穎汝
    - 流動護士： 上次簽章: 張玉梅
    - 時間： 0030

  ,
  - 第二次：
    - 刷手護士 ： 邱權婇/胡穎汝
    - 流動護士： 上次簽章: 張玉梅
    - 時間： 0050

  ,
  - 第三次：
    - 刷手護士 ： 邱權婇
    - 流動護士： 胡穎汝
    - 時間： 0100

備註

備註： NIL

- 記錄者： 上次簽章: 張玉梅
- 時間： 2230

填寫護理人員 胡穎汝

正式摘要

	W52 / W52	

doctorRank:B
